# Quantitative assessment of atrial conduit function: a new index of diastolic dysfunction

**DOI:** 10.1007/s00392-015-0882-8

**Published:** 2015-06-30

**Authors:** Rosaria Nappo, Anna Degiovanni, Virginia Bolzani, Chiara Sartori, Gabriella Di Giovine, Paolo Cerini, Rita Fossaceca, Sándor J. Kovács, Paolo N. Marino

**Affiliations:** Clinical Cardiology, Department of Translational Medicine, Azienda Ospedaliero Universitaria “Maggiore della Carità”, Università del Piemonte Orientale, Corso Mazzini 18, 28100 Novara, Italy; Radiology, Department of Translational Medicine, Azienda Ospedaliero Universitaria “Maggiore della Carità”, Università del Piemonte Orientale, Corso Mazzini 18, 28100 Novara, Italy; Cardiovascular Biophysics Laboratory, Washington University Medical Center, 660 South Euclid Avenue, St Louis, MO 63110 USA

**Keywords:** Diastolic dysfunction, Left atrial conduit function, Full-volume 3D-echocardiography

## Abstract

**Background:**

Heart failure (HF) epidemic has increased need for accurate diastolic dysfunction (DD) quantitation. Cardiac MRI can elucidate left atrial (LA) phasic function, and accurately quantify its conduit contribution to left ventricular (LV) filling, but has limited availability. We hypothesized that the percentage of LV stroke volume due to atrial conduit volume (LACV), as assessed using 3D-echocardiography, can differentiate among progressive degrees of DD in HF patients.

**Methods and results:**

Sixty-three subjects (66 ± 12 years) with DD and ejection fraction (EF) ranging 14–62 % underwent full-volume 3D-echocardiography. Simultaneous LA and LV volume curves as function of time (*t*) were calculated, with LACV as ${\text{LACV}}\left( t \right) \, = \, \left[ {{\text{LV}}\left( t \right){-}{\text{LV minimum}}} \right] \, - \, \left[ {{\text{LA maximum }} - {\text{ LA}}\left( t \right)} \right]$, expressed as % of stroke volume. Patients were assigned to four (0–3, from none to severe) DD grades, according to classical Doppler parameters. In this population DD is linked to LACV, with progressively higher percentages of conduit contribution to stroke volume associated with higher degrees of DD (*p* = 0.0007). Patients were then dichotomized into no-mild (*n* = 26) or severe (*n* = 37) DD groups. Apart from atrial volume, larger (*p* < 0.02) in severe DD group, no differences between groups were found for LV diastolic and stroke volume, EF, mass and flow propagation velocity. However, a significant difference was found for LACV expressed as % of LV stroke volume (29 ± 15 vs. 43 ± 23 %, *p* = 0.016).

**Conclusions:**

Our study confirms that LACV contribution to stroke volume increases along with worsening DD, as assessed in the context of (near) constant-volume four-chamber heart physiology. Thus, LACV can serve as new parameter for DD grading severity in HF patients.

## Introduction

Diastolic dysfunction (DD) is a common medical condition whose identification and grading may be difficult using only a traditional noninvasive approach [1]. Making use of both classical Doppler parameters and left atrial (LA) functional data may be important in this regard, as such integration can contribute to a correct interpretation of the Doppler ventricular filling profile in the “puzzling” patient [2].

Previously, an approach that integrated the information derived from the Doppler mitral flow velocity curve with that of one single pulmonary vein (taken as a representative of all the four veins) allowed us to investigate, quantitatively, the changes in atrial reservoir, pump and conduit function that are associated with increasing degrees of left ventricular (LV) filling impairment [3]. In that study, we demonstrated an inverse linear relation between the conduit contribution to LV filling volume and a “classical” diastolic descriptor, i.e., the Doppler E-wave deceleration time [3, 4]. The approach we used, however, involved several assumptions and did not allow us to delve further into the potential role of atrial conduit function itself as an index of diastolic function. The conduit delivered volume of blood entering the LV cannot be easily separated from the overall early rapid filling volume (E-wave volume) of the ventricle.

**Fig. 1:**
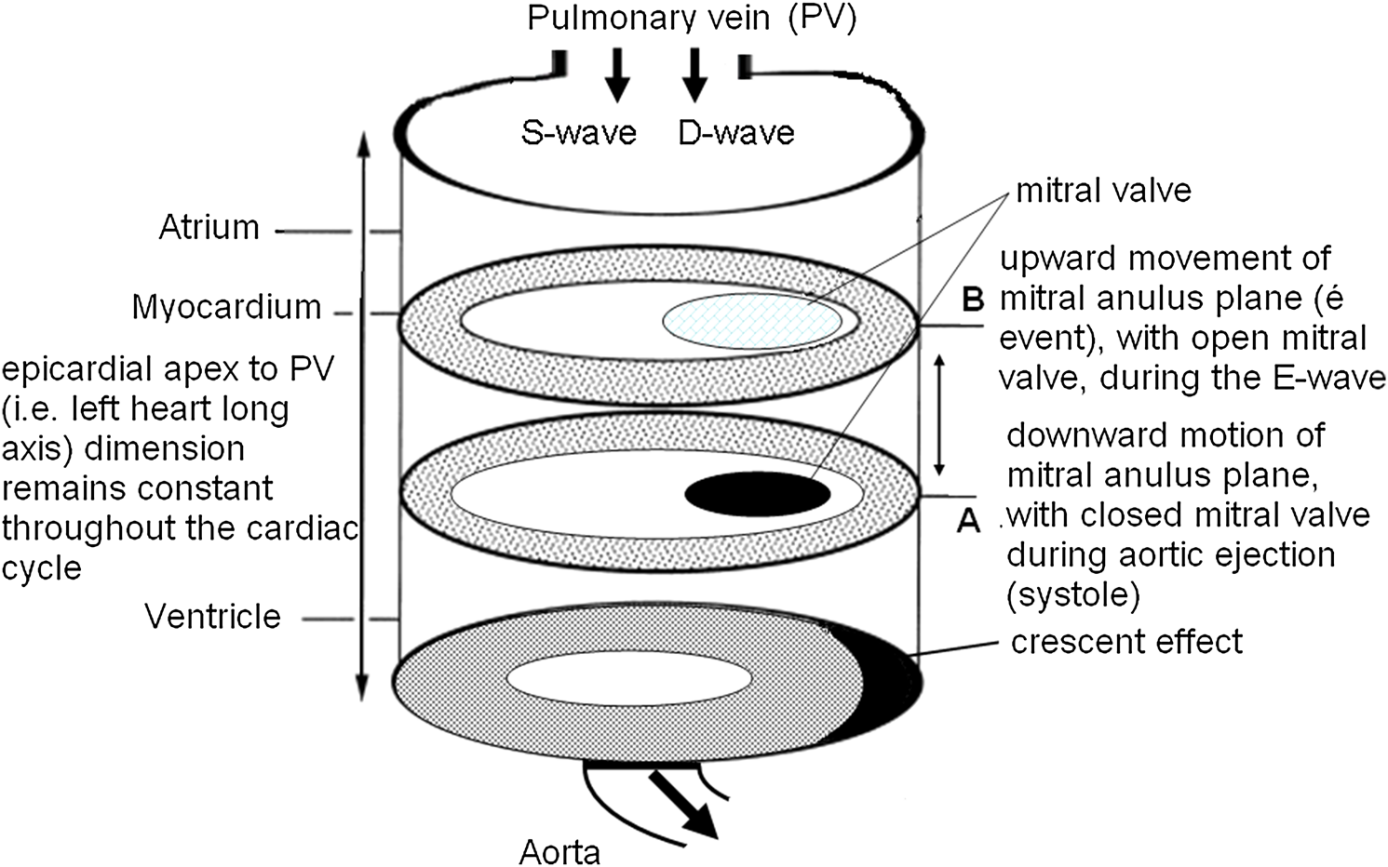
Simplified schematic of left heart illustrating mechanism of left atrial conduit volume (LACV) generation during diastole. Events of the cardiac cycle. **a** At end-systole the ventricle has ejected its stroke volume into the aorta, while simultaneously the mitral annulus has descended and the pulmonary vein S-wave has been generated by aspirating blood from the lung due to the descent of the anulus plane and closed mitral valve (atrial reservoir function). During systole the ventricular epicardial surface has also moved slightly (radially) inward, generating the “crescent effect”, compared to its end-diastolic configuration. **b** In diastole the mitral annulus ascends with open mitral valve. E-wave is initiated by ventricular suction and pulmonary vein D-wave (atrial conduit function) is simultaneously inscribed as ventricular epicardial surface expands radially to reverse the “crescent effect”. Note: actual atrial volume is decreasing while ventricular volume is increasing, because of ascent of the mitral annulus. For clarity, atrial systole (atrial pump function, Doppler A-wave) is not shown because it is not a determinant of LACV. Volume conservation for the left heart for each cardiac cycle requires that: (volume leaving the left heart through aorta) = (volume entering the left heart through the pulmonary veins). Therefore, (ventricular stroke volume) = (pulmonary vein S − pulmonary vein D − wave volume) or (ventricular stroke volume) − (pulmonary vein D − wave volume) = LACV. Because ventricular stroke volume is always greater than the simultaneously entering pulmonary vein S-wave volume, the difference can only be made up in diastole as the D-wave, whose volume is the volume generated by the “crescent effect”. See Ref. [5] and text for details

Recently, in characterizing the governing role of four-chamber (near) constant -volume pump physiology, wherein the atrial and ventricular volumes simultaneously reciprocate throughout the cardiac cycle, MRI has elucidated and characterized LA and LV phasic function, thereby quantifying the conduit contribution to LV filling as the integral of net, diastolic, instantaneous difference between synchronized atrial and ventricular volume curves [5]. In the simplest terms, because LV systolic ejection volume is greater than the volume that simultaneously enters the LA during systole (pulmonary vein S-wave integral), the difference between ejected and entering volumes during systole is made up in the next diastole, during the E-wave (pulmonary D-wave integral) and defines the atrial conduit volume (LACV) (Fig. 1).

Because cardiac MRI availability is limited, we employed 3D-echocardiography to acquire complete and simultaneous LA and LV full-volume datasets to characterize the volume of both left-sided cardiac chambers at each time point during the cardiac cycle in order to quantify LACV in patients after a recent hospital admission for congestive heart failure (HF). In the present study, in accordance with our previous findings [3], we hypothesized that LACV, expressed as a % of LV stroke volume, would be modest in cases of no or mild DD, but greatly increased with more advanced degrees of DD.

## Methods

After appropriate informed consent in agreement with institutional human review studies committee guidelines and local IRB approval in accordance with the ethical standards laid down in the 1964 Declaration of Helsinki and its later amendments, we enrolled 64 HF patients (48 males) in sinus rhythm, aged 67 ± 12 years, with DD and various degrees of associated systolic dysfunction [ejection fraction (EF) 37 ± 28 %, range 14–62 %), admitted with clinical and radiological evidence of pulmonary congestion, no evidence of major residual valvular incompetence and a usable 3D echocardiographic atrioventricular acquisition (vide infra). They were imaged (Vivid E9, GE Medical System, Horten, Norway) according to ASE criteria [6] during their hospital stay or a few days after discharge (6 ± 13), when no residual signs of overt volume or pressure overload could be reasonably detected.

Patients were assigned to 4 (0–3, from none to severe) DD grades [7], according to classical Doppler parameters (pulsed-wave Doppler recordings of the mitral flow velocity obtained from an apical four-chamber view, assuring the presence of a diastatic interval as a clear separation between Doppler the E- and A-waves), mitral annular early diastolic velocities (*e*’) by pulsed-wave tissue Doppler sampled at both septal and lateral annular aspects and biplane LA volume at end-systole (ES) quantified using an area-length algorithm [8]. They were further characterized with *E*/*A*, mitral deceleration time and *E*/*e*’ (taken as the average between septum and lateral annulus) [7].

**Fig. 2:**
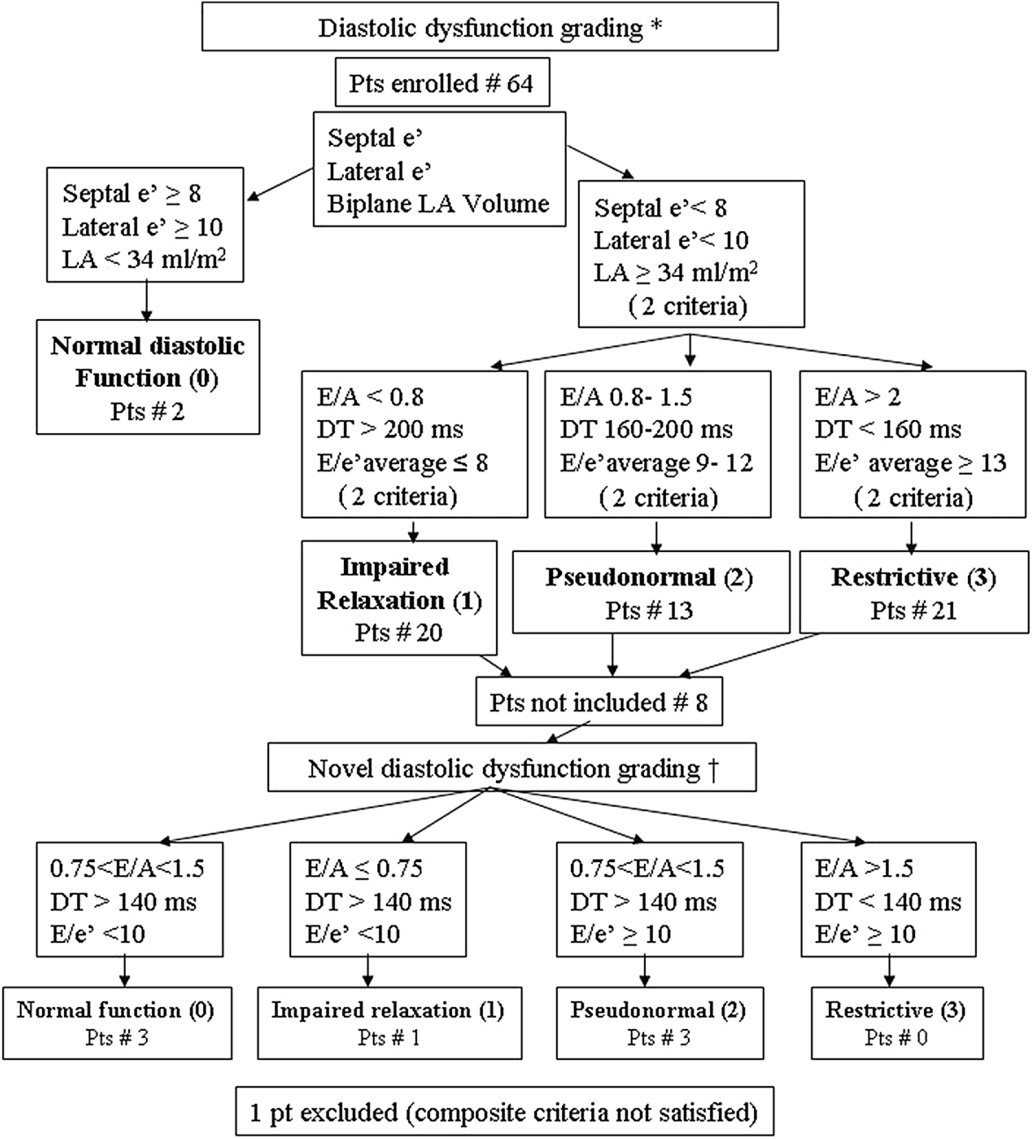
Flow-chart of the diastolic dysfunction grading process. *DT* deceleration time, *LA* left atrial, *Pts* patients. Asterisk denotes Ref. [7] and dagger denotes Ref. [9]

We assigned each patient to a specific DD grade according to the fulfillment of at least two criteria (Fig. 2). Eight patients, who could not be unequivocally classified with current DD grading system, were re-analyzed and attributed to a specific grade according to the recent proposal of Kuway et al. [9].

Color M-mode Doppler mitral flow propagation velocity (*V*_p_), obtained in a four-chamber apical projection, was also available in each patient [10], but not necessary for DD grading purposes.

### Automatic four-dimensional volumetric computation

At the end of the standard examination a multi-beat (at least five consecutive cardiac cycles) pyramidal 3D-echocardiographic full-volume dataset was acquired from the apex in each patient, using a 3 V transducer. The volume data were displayed in real-time: two orthogonal apical views and three cross-sectional slices, with optional volume rendering techniques for visualization of valves and structures were obtained. Full-volume acquisition was generated during held respiration, dataset sector (frame rate ranging between 20 and 40 frames/s) dimensions and depth were set to include both the ventricle and the atrium, assuring volume sampling rates between 15 and 25/s [11].

Subsequently, using a commercially available software package (EchoPAC PC version BT112, GE Healthcare), each view of the averaged single beat was aligned to the standard apical views, using the positions of the mitral annulus plane and the apex as markers. Initialization was done at end-diastole (ED), using two clicks in each view (apex and mitral annulus plane), with contouring and 3D surface detection automatically drawn. Once completed for ED, the same procedure was used for ES, with LV endocardial border tracked throughout the entire cardiac cycle [12]. Surface 3D detection was automatically triggered, and if necessary the detected 3D surface was edited manually by adding landmark points. After both ED and ES images were finalized, full 4D surface detection was performed. Ventricular mass computation was achieved using epicardial border identification at ES, with manual correction as a possible option.

The same procedure was performed for the LA chamber using the mitral annular plane and the roof of the cavity as reference markers [13]. Finally, LV and LA volumes, along with their respective time-volume curves, were derived from the triangulated surfaces by summation of all triangular patches using the divergence theorem [13].

**Fig. 3:**
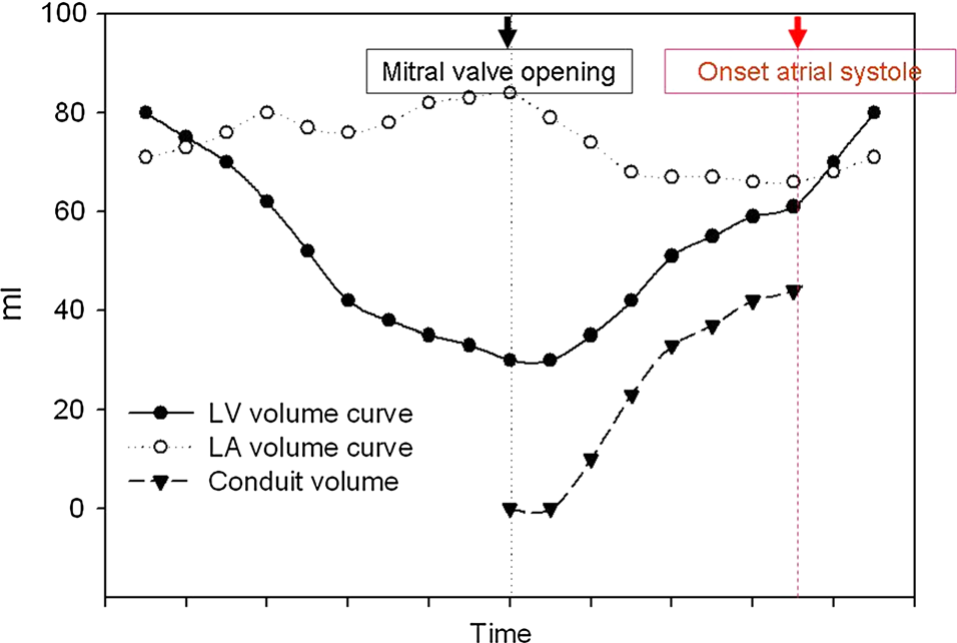
Study output. Continuous ventricular, atrial, and conduit volume curves in a given patient. *LA* left atrial, *LV* left ventricular

A commercially available spreadsheet program was used to transfer data and synchronize the LA and LV volume curves as a function of time (*t*) (Fig. 3). LACV was quantified according to the formula: ${\text{LACV}}\left( t \right) \, = \, \left[ {{\text{LV}}\left( t \right) \, {-}{\text{ LV minimum}}} \right] \, - \, \left[ {{\text{LA maximal }} - {\text{ LA}}\left( t \right)} \right]$ [5], integrating volume data from minimum LV volume to the beginning of the atrial contraction (as identified from simultaneously acquired ECG signal) and expressed as percent of LV stroke volume. In the example shown in Fig. 3:

1. During systole, LV volume decreases from 80 to 30 (ejects 50 ml), while LA volume increases from 70 to 85 (fills by 15 ml). This 15 ml constitutes the LA reservoir volume (volume temporarily stored in LA before mitral valve opening).
2. During diastole, the difference between LV ejected and reservoir volume that entered (50 − 15 ml) must flow from pulmonary veins to LA to LV, thus generating LACV (volume directly going through LA).
3. At each time *t* between mitral valve opening and onset of atrial systole, LACV(*t*) is calculated as follows:

1 $${\text{Change in LV volume }} = {\text{ LA outflow }} = {\text{ LV}}\left( t \right) \, {-}{\text{ LV minimum}} .$$

2 $${\text{Change in LA volume }} = {\text{ LA inflow }} - {\text{ LA outflow }} = {\text{ LA}}\left( t \right) \, {-}{\text{ LA maximal}} .$$

Adding Eqs. 1 and 2 yields

$${\text{LA inflow }} = \, \left[ {{\text{LV}}\left( t \right) \, {-}{\text{ LV minimum}}} \right] \, - \, \left[ {{\text{LA maximal }} - {\text{ LA}}\left( t \right)} \right].$$

### Speckle tracking imaging technique

Using the same dataset a 3D strain tracking technique was also performed starting from the same region of interest (ROI), although not limited to the ventricle and integrated as the last step in the “ventricular quantification tool”, which includes volume and mass measurements [14]. The meshes created for these two measurements were re-used for the LV and LA 3D strain computation. The 3D strain ROI was automatically generated in the ED frame and was built up from the endocardial and the epicardial meshes. The ventricular endocardial mesh was based on the previously used ED volume measurement. The epicardial mesh was automatically generated from the epicardial mesh used during the LV mass determination step, by propagating it from ED to ES.

A display that allowed visual inspection of tracking correctness was used, so that the operator could manually approve or reject the results for individual segments, with segments with poor tracking being removable from the calculation of the global value. From the tracking process transmural strains could be generated for individual segments (*n* = 17) obtained from three apical (longitudinal strain) and/or three short-axis slices (circumferential strain). For the purposes of this study, apical strains (five segments) were excluded from the final computation, while strains, in the longitudinal and circumferential direction (six segments each), were averaged at basal and mid ventricular level slice using custom software.

The same process was applied to atrium, excluding the apical strains (five segments) corresponding to the roof of the cavity. Strains, in the longitudinal and circumferential direction (six segments each), were also averaged at basal and mid atrial slice level, similarly to what had been done for the ventricle.

Strain curves were exported as digital matrix data to a proprietary, open source analysis software (StrATo version 2.0.3.0).

### Cardiac MRI acquisition

Methodological validation was performed in eight further hypertensive HF subjects (70.9 ± 6.4 years old, 2 males), not included in the original population but with compatible LV volumetric and filling characteristics (ED volume 91.3 ± 21.3 ml/m^2^, *E*/*A* ratio 0.81 ± 0.12, *E*/*e*’ 9.1 ± 1.4) but preserved EF (62 ± 5 %, range 51–68 %), who underwent a complete cardiac MRI study and a concurrent 3D echocardiographic examination. All patients were hemodynamically stable and had provided written, informed consent. MRI was performed with a 1.5-T scanner (Philips Medical System, Intera Achieva, Best, The Netherlands) using front and back surface coils and retrospective ECG triggering to capture the entire cardiac cycle. All MRI scans were acquired by the same operator. Survey images and standard planes for ventricular four-chamber and short-axis views were obtained. From the high resolution four-chamber cine loops, obtained during subjects’ breathholds and divided into 30 cardiac phases triggered from the ECG QRS, we planned a short-axis cine stack protocol of 20 slices spanning the apex of the ventricle through the superior posterior wall of the atrium. The data were archived and transferred to a remote workstation to perform off-line analysis. In every short-axis slice endocardial boundaries were traced manually during the ED and ES phases in order to obtain the LV and LA volumetric parameters via the section summation method. The free papillary muscles were excluded from LV volume assessment. In analysis of echo data the LA appendage was excluded from LA volume measurement.

### Statistics

Data are expressed as mean 1 ± SD. Differences between means were assessed by unpaired *t* tests. A Mann–Whitney rank sum test was used if data were not normally distributed. Correlation analysis was used to assess the relation between conduit function, DD data, and atrial and ventricular parameters by comparing Spearman’s rank order correlation coefficients. Least square regression analysis was used as necessary. A backward stepwise regression model was also developed in order to identify which diastolic function variables, as measured within the study, besides age and BMI, predicted DD grading in our patients’ population, with conduit, as a potential predictor, forced into the model.

To find a diagnostic conduit function cut-off value for identification of severe vs. no or mild (see below) degree of DD, nonparametric receiver-operating characteristics (ROC) curve analysis was performed and the area under the curve showing the discriminatory ability of the variable cut-off was reported. Sensitivity and specificity values of the best cut-off variable were also calculated, while the method of DeLong, DeLong and Clarke-Pearson was used to compare areas.

Finally, a two-way repeated-measures ANOVA was used to assess the effects of ventricular and atrial level (basal vs. mid) on strain values, with the attribution to conduit categories (see below) groups as a between-patient factor. The Tukey test was used for pairwise multiple comparisons. A *p* value < 0.05 was considered to be significant. Statistical analyses were performed using Sigmaplot (version 12.5 for Windows, Jandel, San Rafael, CA, USA).

According to previously published data from our group [3], we assumed that a 20 % difference in conduit function between severe and no or mild degree of DD could be detected with 31 subjects per group, assuming a SD of 20 % with a power = 0.90 and *α* = 0.01.

## Results

**Fig. 4:**
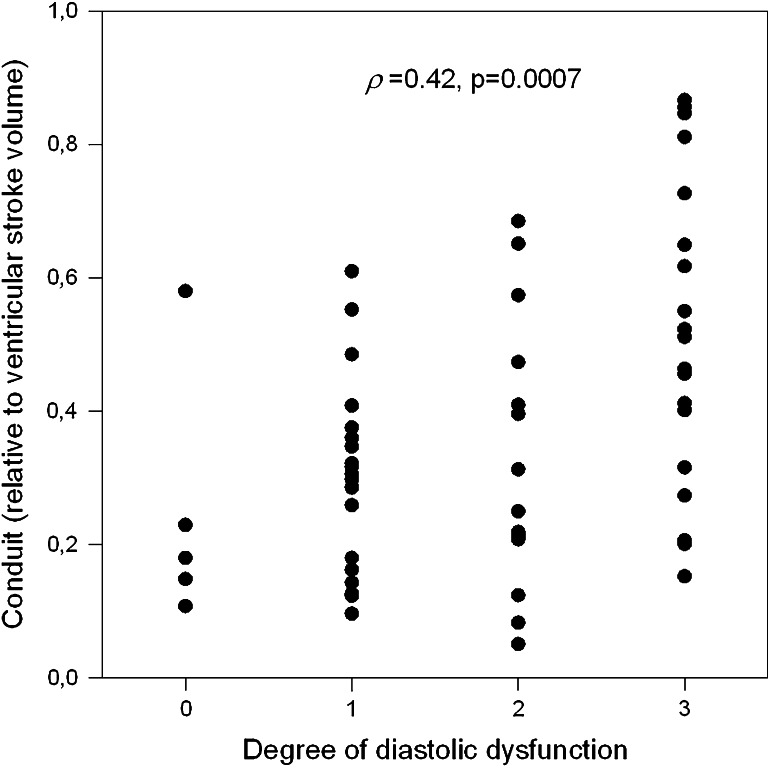
Relation between conduit function and degree of diastolic dysfunction. There is a clear, positive linear relation between no and progressive degrees of ventricular diastolic dysfunction, as assessed using classical Doppler parameters, and conduit function expressed relative to ventricular stroke volume in our patients’ population

The flow-chart describing the attribution of each patient to a given DD class is shown in Fig. 2. One patient could not be attributed and was excluded from analysis. The clinical characteristics of the remaining population are reported in Table 1. In two patients conduit could not be computed due to technical problems (inadequate quality of the recorded images). According to the results of the Spearman’s product moment correlations (Table 2), DD appears, in the remaining population, to be linked to atrial conduit function, with a progressively higher percentage of conduit contribution to LV filling volume associated with higher degrees of DD (*p* = 0.0007, Fig. 4). A weaker, borderline relation (*p* = 0.03) between DD and conduit as flow rate and expressed in ml/s/m^2^ could also be detected. There was an obvious positive significant relation between degrees of DD and LA minimal (*p* = 0.0007) and maximal (*p* = 0.007) volumes (as derived from the continuous LA volume curve) and an inverse relation with LA peak longitudinal strain (*p* = 0.016) and EF (*p* = 0.012). No relation, was observed between DD and LA circumferential strain, *V*_p_, LV volume or mass (*p* = NS for all).

**Table 1:** Characteristics of the patients’ population (*n* = 63)

Gender (M/F)	48/15
Age (years)	66.4 ± 12.2
BMI (kg/m^2^)	25.9 ± 4.4
Body surface area (m^2^)	1.84 ± 0.21
NYHA class	2.2 ± 0.8
Etiology of heart disease (*n*, %)	
HIV-related	1 (1.6)
Idiopathic	33 (52.4)
Ischemic	23 (36.5)
Valvular	6 (9.5)
Drugs at hospital discharge (*n*, %)	
Diuretics	64 (100)
Beta blockers	58 (90.6)
ACE-inhibitors	50 (78.1)
Angiotensin II receptor antagonists	12 (18.7)
Left ventricular end-diastolic volume (ml/m^2^)	87.2 ± 27.3
Left ventricular end-systolic volume (ml/m^2^)	56.4 ± 25.6
Ejection fraction (%)	37.1 ± 11.3
Left ventricular mass (g/m^2^)	79.6 ± 11.5
Mitral flow propagation velocity (cm/s/m^2^)	25.3 ± 6.1
Mitral E-wave (cm/s)	61.9 ± 23.1
Mitral A-wave (cm/s)	66.0 ± 23.9
*E*/*A*	1.1 ± 0.8
Mitral deceleration time (ms)	187.7 + 90.8
*e*’ septum (cm/s)	5.0 ± 2.4
*e*’ lateral wall (cm/s)	6.7 ± 3.6
*e*’ average (cm/s)	5.8 ± 2.5
*E*/*e*’	17.3 ± 25.8
Biplane left atrial end-systolic volume (ml/m^2^)	31.9 ± 14.7
Left atrial conduit function	
% ventricular stroke volume	37.6 ± 21.2
absolute, instantaneous flow rate terms, ml/s/m^2^	58.4 ± 39.2

**Table 2:** Spearman Rank order correlations of diastolic dysfunction grade and echocardiographic parameters

DD	Conduit (%)	Conduit flow rate (ml/s/m^2^)	LA volume max (ml/m^2^)	LA volume min (ml/m^2^)	EF (%)	LA longitudinal strain	LA circumferential strain
Number of patients	61	61	63	63	63	42	42
Correlation coefficient, *ρ*	0.424	0.278	0.340	0.416	−0.317	−0.372	−0.295
*p*	0.0007	0.03	0.006	0.0007	0.012	0.016	0.058

*DD* diastolic dysfunction grade, *EF* ejection fraction, *LA* left atrial, *max* maximum, *min* minimum

The cohort was then arbitrarily dichotomized into no or mild (0–1, *n* = 26) vs. severe (2–3, *n* = 37) DD groups (Table 3). Apart from LA volume, which was significantly larger in the severe DD group (*p* < 0.02 for both minimum and maximum volume) and LA strains, insignificantly higher in no or mild vs. severe DD group, no differences between the two cohorts were found for LV ED, ES and ejected volume, EF, cardiac mass, or *V*_p_ (*p* > 0.05 for all, Table 3). A significant difference between no or mild and severe DD groups was found for LACV, computed as % of LV stroke volume (*p* = 0.016); when conduit, however, was expressed in instantaneous flow rate volume terms the difference did not reach statistical significance (Table 3).

**Table 3:** Characteristics of the population divided according to diastolic dysfunction (DD) grade

	DD no or mild	DD severe	*p*
Gender (M/F)	21/5	27/10	
Age (years)	62.7 ± 11.4	69.1 ± 12.2	0.04
NYHA class	1.9 ± 0.9	2.4 ± 0.7	0.01
Left ventricular end-diastolic volume (ml/m^2^)	83.1 ± 23.6	90.1 ± 29.6	0.47
Left ventricular end-systolic volume (ml/m^2^)	50.5 ± 20.8	60.2 ± 28.1	0.18
Left ventricular stroke volume (ml/m^2^)	32.2 ± 8.7	29.8 ± 9.1	0.29
Ejection fraction (%)	40.1.1 ± 10.4	35.0 ± 11.5	0.08
Left ventricular mass (g/m^2^)	79.0 ± 12.6	79.9 ± 10.8	0.77
Mitral flow propagation velocity (cm/s/m^2^)	26.6 ± 5.7	24.3 ± 6.2	0.16
Left atrial minimum volume (ml/m^2^)	21.2 ± 18.9	30.0 ± 16.6	0.01
Left atrial maximum volume (ml/m^2^)	30.6 ± 18.0	39.0 ± 17.2	<0.02
Left atrial longitudinal strain	0.13 ± 0.10	0.08 ± 0.08	0.085
Left atrial circumferential strain	0.14 ± 0.15	0.09 ± 0.12	0.174
Left atrial conduit function			
% ventricular stroke volume	29.1 ± 15.0	43.4 ± 23.1	<0.02
Instantaneous flow rate (ml/s/m^2^)	52.0 ± 40.6	62.8 ± 38.2	0.09

Using ROC analysis, we identified LACV 39 % of stroke volume as capable of discriminating between no or mild and severe degree of DD (ROC area 0.67, *p* = 0.02), with good sensitivity (80 %) but moderate specificity (61 %). Specificity and sensitivity were 44 and 80 %, respectively, when values 64 ml/s/m^2^ were used as LACV flow rate cut-off (ROC area 0.62, *p* = NS). ROC area value was 0.69 for minimum (*p* < 0.01), 0.68 for maximum (*p* < 0.02) LA volume, and 0.64 for EF (*p* = 0.06). ROC curve areas were not significant for ventricular ED and ejected volumes (0.55, 0.61, respectively), mass (0.52), *V*_p_ (0.60), or LA strains (0.57 and 0.56 for longitudinal and circumferential direction, respectively) (*p* = NS for all).

**Fig. 5:**
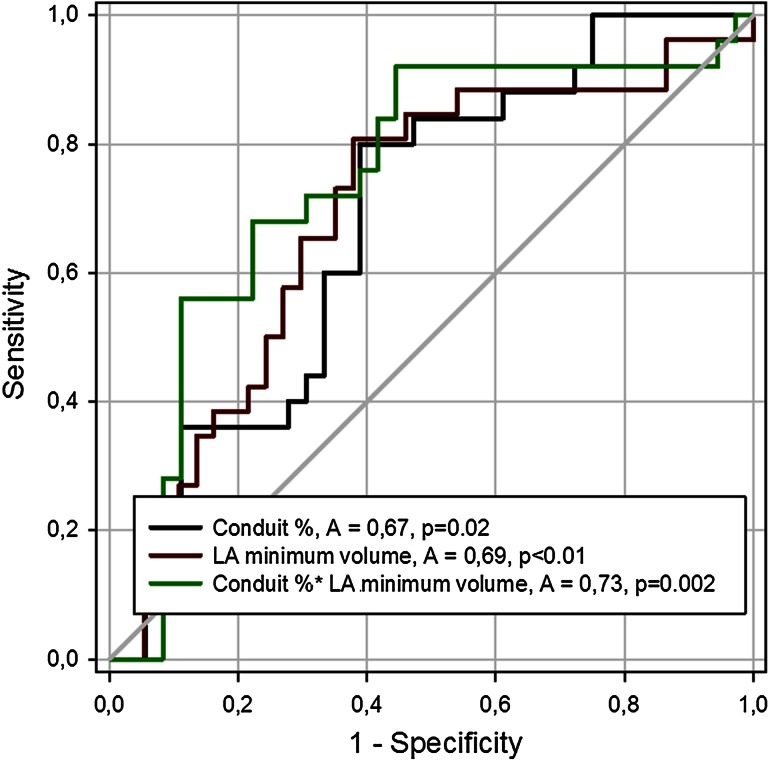
ROC curves for the identification of no or mild vs. severe diastolic dysfunction groups according to various echocardiographic parameters. Using ROC analysis, we identified LACV 39 % of ventricular stroke volume as capable of discriminating between no or mild and severe degree of diastolic dysfunction (ROC area 0.67, *p* = 0.02) with good sensitivity (80 %), although moderate specificity (61 %). For atrial minimum volume sensitivity and specificity were 81 and 62 %, respectively, when 20 ml/m^2^ were used as cut-off value (ROC area 0.69, *p* < 0.01). ROC area value was 0.73 for combined conduit and minimal left atrial volume metrics (*p* = 0.002). No significant differences were detected when comparing areas under the curves. *A* ROC area, *LA* left atrial

There was a weak correlation between LACV, expressed as % of LV stroke volume, and LA minimum volume (*r* = 0.28, *p* < 0.03). Their product (LACV × LA minimum volume), viewed as a reflection of combined LV and LA metrics, provided the largest ROC curve area with a value of 0.73 (*p* = 0.002, Fig. 5). Sensitivity and specificity (92 and 56 % for a value of 787 % ml/m^2^), however, did not differ statistically from what obtainable from LACV or LA minimum volume (81 and 62 % for a value of 20 ml/m^2^), alone.

Rearranging LACV according to its median value (32 %), we were able to demonstrate its significant association with the majority of the diastolic function indexes used throughout the manuscript (Table 4). Such association was much less strong (quantitatively and qualitatively) for LA maximal volume (data not shown). Only minimal LA volume (median 19.08 ml/m^2^) demonstrated stronger associations with these same diastolic parameters (Table 4). It has to be underlined, however, that such association was against what expected for LV mass, where a negative, counterintuitive, significant correlation was detectable with LA minimum volume (Table 4). The same results, for both conduit and LA volumes could be obtained if full range data, instead of categorical ones, were used.

**Table 4:** Spearman Rank order correlations of conduit, computed as % of left ventricular (LV) stroke volume, and left atrial (LA) minimum volume, both categorized according to their respective medians (≤32 % and ≤19.08 ml/m^2^), and the diastolic function indexes used throughout the manuscript

	Mitral E-wave (cm/s)	*E*/*A*	Deceleration time (ms)	*e*’ average (cm/s)	*E*/*e*’	Mitral flow propagation velocity (cm/s/m^2^)	LV mass (g/m^2^)
Conduit							
Number of patients	60	54	57	46	46	57	61
Correlation coefficient, *ρ*	0.364	0.399	−0.304	−0.145	0.378	−0.0811	0.102
*p*	0.0044	0.00292	0.0217	0.336	0.0098	0.547	0.431
LA minimum volume							
Number of patients	62	55	59	48	48	59	63
Correlation coefficient, *ρ*	0.549	0.634	−0.462	−0.0317	0.302	−0.146	−0.313
*p*	0.000005	0.000001	0.00026	0.830	0.0369	0.267	0.0128

The backward stepwise regression analysis demonstrated that LACV, computed as % of LV stroke volume, was a significant independent predictor of DD grading (*β* = 0.267 *p* = 0.046), ranking third after *E*/*A* ratio (*β* = 0.546, *p* < 0.001) and *e*’ (*β* = −0.295, *p* = 0.025). None of the other variables listed in Table 4, including age and BMI, predicted DD grading in our patients’ population.

### Relation between conduit function and bidirectional ventricular strain behavior

The values of averaged ventricular longitudinal and circumferential strains, measured at basal and mid ventricular level and arranged according to the median value of conduit (32 %) are reported in Table 5. There is no difference of averaged longitudinal strains with increasing values of conduit, basal values averaging less as compared with those measured at mid ventricular level (*p* = 0.003 for trend).

**Table 5:** Longitudinal and circumferential ventricular and atrial strains measured at basal and mid cavity slice level and arranged according to the median (32 %) of atrial % conduit contribution to ventricular stroke volume

Conduit contribution to ventricular stroke volume	≤32 %	>32 %	2-ANOVA with repeated measurements
Basal ventricular longitudinal strain	−0.09 ± 0.03	−0.08 ± 0.03	Interaction effect, *p* = 0.62
Mid ventricular longitudinal strain	−0.11 ± 0.03	−0.10 ± 0.04	Slice level effect, *p* = 0.003
Basal ventricular circumferential strain	−0.10 ± 0.04	−0.11 ± 0.05*	Interaction effect, *p* = 0.021
Mid ventricular circumferential strain	−0.10 ± 0.05	−0.08 ± 0.04	Slice level effect, *p* = 0.002
Basal atrial longitudinal strain	0.12 ± 0.11	0.06 ± 0.07	Slice level effect, *p* = 0.87
Mid atrial longitudinal strain	0.12 ± 0.09	0.07 ± 0.09	Conduit effect, *p* = 0.044
Basal atrial circumferential strain	0.15 ± 0.14	0.08 ± 0.11	Slice level effect, *p* = 0.002
Mid atrial circumferential strain	0.09 ± 0.09	0.06 ± 0.07	Conduit effect, *p* = 0.09

*p < 0.001 vs. mid ventricular circumferential strain according to Tukey test

Values for ventricular circumferential strains are also shown in Table 5. For the entire population circumferential strain averaged −0.11 ± 0.05 at basal level, a value greater than that computed at the mid short-axis slice (−0.09 ± 0.04, *p* = 0.002). At a difference, however, with longitudinal strain, ventricular circumferential strain at basal level increased with increasing degrees of conduit, while it decreased at mid level (interaction *p* = 0.021) so that, for large conduit contribution to LV filling volume, circumferential strain averaged −0.11 ± 0.05 at basal as compared with −0.08 ± 0.04 at mid ventricular level (*p* < 0.001).

### Relation between conduit function and atrial strain behavior

The values of average circumferential strain, measured at basal and mid atrial level and arranged according to the median value of conduit (2 %) are reported in Table 5. There is no difference of circumferential strains with increasing values of conduit, basal values averaging more as compared with those measured at mid atrial level (*p* = 0.002).

Values for atrial longitudinal strains are also reported in Table 5. Similarly to the behavior of the ventricle, averaged longitudinal atrial strains decreased with increasing degrees of conduit (*p* < 0.05), but with no difference between levels.

These results indicate that increments in the conduit phasic function in DD patients are associated with a progressive decline in longitudinal strains at both atrial and ventricular level, compensated by deformation in the circumferential direction detectable only at the base of the ventricle [15].

### Comparison between cardiac MRI and 3D echocardiography in assessing conduit volume

The mean absolute (i.e., without ± sign) difference between the two techniques in quantifying conduit volume averaged 4.1 ± 3.2 ml. The equivalent value for conduit expressed relative to LV stroke volume was 7 ± 5 %. In particular the mean value for conduit expressed in absolute or percentage values for MRI was 20.2 ± 5.1 ml and 39 ± 8 % and for 3D echocardiography 18.6 ± 8.2 ml and 36 ± 12 %, respectively (*p* = NS for both). Alternatively, the correlation coefficients between the two measurements was 0.83 (*p* = 0.012) for conduit quantified in absolute terms and 0.72 (*p* = 0.04) when expressed in percent. A plot of the average between the two techniques against their difference demonstrated no over/underestimation for 3D echocardiography vs. MRI, with a comparable dispersion of the data for conduit computed relative to stroke volume (limits of agreement: from −19.9 to +13.6 %) as compared with conduit expressed in absolute terms (limits of agreement: from −11.9 to +8.7 ml). There was, however, no relation between the two techniques when conduit was quantified in instantaneous flow rate terms.

### Reproducibility

Interobserver variability for conduit, determined from nine randomly selected patients and expressed as absolute mean difference ± the percentage coefficient of variation (SD/mean) was 4.5 ml ± 1.3 % or 15.0 ml/s/m^2^ ± 0.9 %.

## Discussion

In this study, our data shows that a direct, clear relation exists between the atrial conduit contribution to ventricular filling volume (expressed either as a percentage of stroke volume or in maximum flow rate terms) and the degree of LV DD in a group of HF patients with various degrees of associated systolic dysfunction.

### Conduit and diastolic dysfunction

Transmitral flow (Doppler E-wave) is determined by the diastolic atrioventricular pressure gradient, whose “upstream” component, represented by the atrial pressure (LAP) [16], is not routinely measured. As DD develops LAP gradually increases, generating the “pseudonormal pattern” in Doppler echocardiography. As a result of DD, the atrial tissue can remodel so that, when atrioventricular stiffening develops, pressure in the atrium will fall very rapidly as the atrial chamber empties in response to LV aspiration of atrial blood to generate the E-wave [17, 18]. Conduit flow from the pulmonary reservoir during diastole assures cyclic restoration of four-chamber volume to its pre systolic level, the LA cavity behaving as a constant pressure source for LV filling due to a combination of conduit and capacitance function [19, 20]. Such considerations support interpreting atrial conduit phasic function as a component of stroke volume in conditions of increased LV resistance to filling and support the expectation that the conduit volume contribution to filling (in normal sinus rhythm) will increase with worsening DD.

Conduit flow commences when mitral valve opens and terminates before atrial contraction [21]. Mechanical recoil of the LV is the energy source that initiates the E-wave and the simultaneous Doppler D-wave through the pulmonary veins. Transmitral flow is modulated by LAP and because conduit volume is effectively aspirated into the ventricle while (anatomic) atrial volume is decreasing due to ascent of the mitral annulus (the back of the atrium remains fixed in the mediastinum and the LV epicardial apex remains fixed in the thorax), the atrium functions as a passive conduit. Accordingly such phasic function may be more appropriately viewed as an LV diastolic function attribute, rather than reflecting intrinsic atrial characteristics [22, 23].

**Fig. 6:**
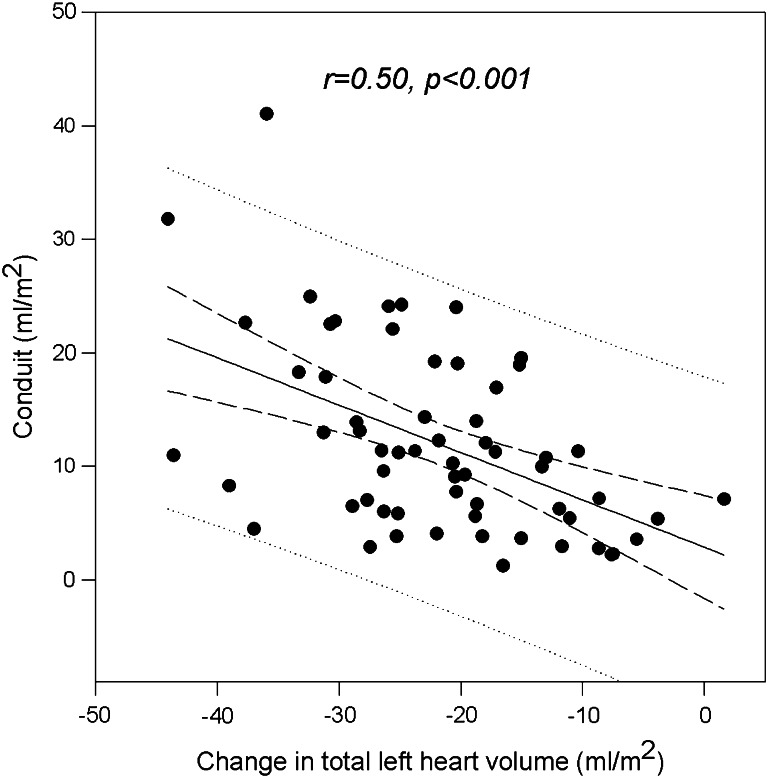
Linear inverse relation between changes in total left heart volume and integrated conduit volume. The amount by which ventricular systole reduces the total left heart volume, relative to the volume simultaneously entering the left atrium, will determine how much blood must flow through the atrium during diastole directly into the ventricle (conduit flow) to make up the difference. Confidence and prediction interval lines are also shown

Our study indicates that because DD directly involves the ventricle, atrial conduit function must change in response in accordance with the constraints of four-chamber (near) constant-volume pump physiology. Data showed the expected inverse relation between the difference in total left heart volume (obtained summing ventricular and atrial volume data) from diastole (at QRS complex time) to systole (at minimum ventricular volume) and conduit volume expressed in absolute terms (*r* = 0.50, *p* < 0.001, Fig. 6). These findings justify the introduction of LACV as a potential index of diastolic function suitable for grading the presence and severity of DD in HF patients. The role of this index may be enhanced in a clinical setting where DD cannot be unambiguously defined like, for example, in patients with chronic hypertension not experiencing signs or symptoms, but known to be at increased risk for HF [24].

### Conduit function in relation to ventricular and atrial strains’ behavior

In our population, we noticed that, at basal level, ventricular circumferential strain increases (gets more negative) with larger amounts of conduit flow, albeit decreasing at mid ventricular level (interaction *p* = 0.021, Table 5).

Such finding is not unexpected. The deviation from the constant-volume rule of the four-chambered heart dictates the “crescent effect”, which implies that the entering conduit volume would be primarily accommodated by some lateral displacement of the epicardial contour of the LV, most often involving the segment not facing the ventricular septum [15, 25] (Fig. 1). Because “longitudinal impedance” of the normal LV during the E-wave is about 34 times lower than “transverse impedance” [26], longitudinal volume accommodation is preferred and is achieved through longitudinal (long axis) displacement of the mitral valve plane. An important characteristic encountered in HF is decreased longitudinal excursion of the mitral valve plane. To maintain stroke volume the compensatory change in epicardial (radial) motion of the heart throughout the cardiac cycle, in fact, manifests in the basal short-axis plane, with minimal variation in the longitudinal direction (Table 5).

In our study, we found an inverse linear relation between conduit volume and circumferential strain measured at the base of the heart (*r* = −0.25, *p* = 0.058). No relation could be detected for longitudinal strain at the same cardiac slice (*r* = 0.17, NS), or for both circumferential and longitudinal strains measured at mid ventricular level (*p* = 0.89 and *p* = 0.61, respectively, NS for both). This independently reinforces the interpretation that conduit volume determines the “crescent effect” and the latter can be detected by circumferential strains [25].

Also atrial longitudinal strains were more depressed as conduit contribution to ventricular stroke volume increased in our patients (*p* = 0.04, Table 5). Such finding is consistent with the recent notion that deterioration in LA function, as quantified using longitudinal strain, mirrors the development of ventricular dysfunction, as recently shown in the MESA study [27]. No such statistically comparable effect was detectable when circumferential strains were considered, although data suggested of a significant difference between basal and mid slice level circumferential deformation (*p* = 0.002 for trend, Table 5).

## Limitations

Because constant-volume pump physiology requires that conduit contribution reciprocate with reservoir and pump function [3, 21], we did not quantitate reservoir and pump function, although we recognize that changes in atrial pump characteristics have been described in patients with DD [28].

The number of patients enrolled into the study was calibrated for detecting a 20 % difference in conduit function between severe and no or mild degree of DD. It has to be acknowledged, however, that our collapsing four DD grades into two categories, because of such planning, has no pathophysiological legitimation.

LA minimum volume and reservoir function have been previously correlated with LV diastolic function [11]. Systolic LV function is a direct determinant of reservoir function, thus limiting its diagnostic potential [29, 30]. Atrial minimum volume may be considered alternative metrics.

It has been reported that atrial conduit function deteriorates with normal aging [31, 32]. Patients’ age ranged from 35 to 90 years in our study, and thus a potential confounding effect of age on indices of LA morphology and function is possible. It has to be pointed out, however, that (1) our study was carried out in patients already diagnosed with congestive HF where the age “effect” may be attenuated, and (2) in those studies conduit had been derived from the continuous LA volume curve only. We know that computing LACV using such a method questions the conduit computation, leading investigators to conclusions that may conflict with results obtained using methods better linked to basic physiology [33]. Finally, (3) age resulted not as a DD predictor according to the multivariate analysis.

LA wall thickness is influential in speckle tracking and strain analysis, and thus it may have had some impact on strain, as assessed in our study [34, 35]. We must acknowledge such limitation that allowed us to obtain atrial strain data only in a fraction (67 %) of our patients’ population.

Finally, low frame rates of 3D echocardiographic assessment of LV and LA volume curves can make the assessment of phasic changes in LA function questionable. We are confident, however, that the sampling rate of the technique (lower limit 15 Hz) should be high enough to describe accurately most of the cavity movements. According to Gibson and Brown, in fact, 10 Hz is the frequency response required to describe most of the motion of the cardiac structures, with only 1 % of the information being present above 30 Hz [36].

## Conclusion

Our work confirms that in response to ventricular DD atrial conduit function is predictably altered in quantifiable ways. Our results suggest that LACV has the potential to serve as a novel index in the categorization and grading of DD and provides a sensitive although moderately specific parameter to differentiate between no or mild and severe forms of DD. In presence of inconsistencies among the various traditionally used indexes (*E*, *e*’, *E*/*A*, and *E*/*e*’ ratio, mitral deceleration and flow propagation velocity, LV mass, and LA volume) [7], computation of LACV can be viewed as a potentially discriminating parameter.

## References

[1] Dragulescu A, Mertens L, Friedberg MK (2013). Interpretation of left ventricular diastolic dysfunction in children with cardiomyopathy by echocardiography: problems and limitations. Circ Cardiovasc Imaging.

[2] Yellin E (1999). Concepts related to the study of diastolic function: a personal commentary. J Cardiol.

[3] Prioli A, Marino P, Lanzomi L, Zardini P (1998). Increasing degrees of left ventricular filling impairment modulate left atrial function in humans. Am J Cardiol.

[4] Marino P, Little WC, Rossi A, Barbieri E, Anselmi M, Destro G (2002). Can left ventricular diastolic stiffness be measured noninvasively?. J Am Soc Echocardiogr.

[5] Bowman AW, Kovács SJ (2004). Left atrial conduit volume is generated by deviation from the constant-volume state of the left heart: a combined MRI-echocardiographic study. Am J Physiol Heart Circ Physiol.

[6] Lang RM, Badano LP, Tsang W, Adams DH, Agricola E, Buck T (2012). EAE/ASE recommendations for image acquisition and display using three-dimensional echocardiography. Eur Heart J Cardiovasc Imaging.

[7] Nagueh SF, AppletonCP Gillebert TC, Marino PN, Oh JK, Smiseth OA (2009). Recommendations for the evaluation of left ventricular diastolic function by echocardiography. Eur J Echocardiogr.

[8] Lang RM, Bierig M, Devereux RB, Flachskampf FA, Foster E, Pellikka PA (2006). Recommendations for chamber quantification. Eur J Echocardiogr.

[9] Kuwaki H, Takeuchi M, Chien-Chia WuV, Otani K, Nagata Y, Hayashi A (2014). Redefining diastolic dysfunction grading. J Am Coll Cardiol Img.

[10] Garcia MJ, Palac RT, Malenka DJ, Terrell P, Plehn JF (1999). Color M-mode Doppler flow propagation velocity is a relatively preload-independent index of left ventricular filling. J Am Soc Echocardiogr.

[11] Russo C, Zhezhen J, Homma S, Rundek T, Elkind MSV, Sacco RL (2012). Left atrial minimum volume and reservoir function as correlates of left ventricular diastolic function: impact of left ventricular systolic function. Heart.

[12] Hansegard J, Urheim S, Lunde K, Malm S, Rabben SI (2009). Semi-automated quantification of left ventricular volumes and ejection fraction by real-time three-dimensional echocardiography. Cardiovascular Ultrasound.

[13] Goldman RN, Arvo J (2004). Area of planar polygons and volume of polyhedra. Graphics gems II.

[14] Jasaityte R, Heyde B, Ferferieva V, Amundsen B, Barbosa D, Loeckx D (2012). Comparison of a new methodology for the assessment of 3D myocardial strain from volumetric ultrasound with 2D speckle tracking. Int J Cardiovasc Imaging.

[15] Waters EA, Bowman AW, Kovács SJ (2005). MRI-determined left ventricular “crescent effect”: a consequence of the slight deviation of contents of the pericardial sack from the constant-volume state. Am J Physiol Heart Circ Physiol.

[16] Ishida Y, Meisner JS, Tsujioka K, Gallo JI, Yoran C, Frater RWM (1986). Left ventricular filling dynamics: influence of left ventricular relaxation and left atrial pressure. Circulation.

[17] Suga H (1974). Importance of atrial compliance in cardiac performance. Circ Res.

[18] Shmuylovich L, Kovács SJ (2007). E-wave deceleration time may not provide an accurate determination of left ventricular chamber stiffness if left ventricular relaxation/viscoelasticity is unknown. Am J Physiol Heart Circ Physiol.

[19] Yellin EL, Dagianti A, Feigenbaum H (1993). Influence of preload, afterload, and contractility on indexes of diastolic function. Echocardiography 1993.

[20] Marino P, Faggian G, Bertolini P, Mazzucco A, Little WC (2004). Early mitral deceleration and left atrial stiffness. Am J Physiol Heart Circ Physiol.

[21] Leite-Moreira AF, Oliveira SM, Marino P (2007). Left atrial stiffness and its implications for cardiac function. Future Cardiol.

[22] Bowman AW, Kovács SJ (2003). Assessment and consequences of the constant-volume attribute of the four-chambered heart. Am J Physiol Heart Circ Physiol.

[23] Hoffman EA, Ritman EL (1985). Invariant total heart volume in the intact thorax. Am J Physiol Heart Circ Physiol.

[24] Lam CS, Roger VL, Rodeheffer RJ, Bursi F, Borlaug BA, Ommen SR (2007). Cardiac structure and ventricular-vascular function in persons with heart failure and preserved ejection fraction from Olmsted County, Minnesota. Circulation.

[25] Riordan MM, Kovács SJ (2006). Relationship of pulmonary vein flow to left ventricular short-axis epicardial displacement in diastole: model-based prediction with in vivo validation. Am J Physiol Heart Circ Physiol.

[26] Axel L (2004). Assessment of pericardial disease by magnetic resonance and computed tomography. J Magn Reson Imaging.

[27] Habibi M, Chahal H, Opdahl A, Gjesdal O, Helle-Valle TM, Heckbert SR (2014). Association of CMR-measure LA function with heart failure development. J Am Coll Cardiol Img.

[28] Melenovsky V, Borlaug BA, Rosen B, Hay I, Ferruci L, Morell CH (2007). Cardiovascular features of heart failure with preserved ejection fraction versus nonfailing hypertensive left ventricular hypertrophy in the urban Baltimore community: the role of atrial remodeling/dysfunction. J Am Coll Cardiol.

[29] Ersbøll M, Andersen MJ, Valeur N, Mogensen UM, Waziri H, Møller JE (2013). The prognostic value of left atrial peak reservoir strain in acute myocardial infarction is dependent on left ventricular longitudinal function and left atrial size. Circ Cardiovasc Imaging.

[30] Appleton CP, Kovács SJ (2009). The role of left atrial function in diastolic heart failure. Circ Cardiovasc Imaging.

[31] Nikitin NP, Witte KK, Thackray SD, Goodge LJ, Clark AL, Cleland JG (2003). Effect of age and sex on left atrial morphology and function. Eur J Echocardiogr.

[32] Okamatsu K, Takeuchi M, Nakai H, Nishikage T, Salgo IS, Husson S (2009). Effects of aging on left atrial function assessed by two-dimensional speckle tracking echocardiography. J Am Soc Echocardiogr.

[33] Marino P (2010). Correct estimation of conduit function from left atrial volume curve assessment only is unlikely. J Am Soc Echocardiogr.

[34] Wang K, Ho SY, Gibson DG, Anderson RH (1995). Architecture of atrial musculature in humans. Br Heart J.

[35] Altekin RE, Yanikoglu A, Karakas MS, Ozel D, Kucuk M, Yilmaz H (2012). Assessment of left atrial dysfunction in obstructive sleep apnea patients with the two dimensional speckle-tracking echocardiography. Clin Res Cardiol.

[36] Gibson DG, Brown DJ (1975). Measurement of peak rates of left ventricular wall movement in man. Comparison of echocardiography with angiography. Br Heart J.

